# Effects of household solid fuel use on sarcopenia in middle-aged and older adults: evidence from a nationwide cohort study

**DOI:** 10.3389/fpubh.2024.1337979

**Published:** 2024-10-02

**Authors:** Shaohui Su, Yinuo Zhou, Kerui Wang, Aonan Liu, Lei Lei, Hao Ma, Yanfang Yang

**Affiliations:** Department of Epidemiology and Biostatistics, West China School of Public Health and West China Fourth Hospital, Sichuan University, Chengdu, Sichuan, China

**Keywords:** sarcopenia, solid fuel use, indoor air pollution, cohort study, CHARLS

## Abstract

**Background:**

Household solid fuel use is common in global households and has been linked to changes in handgrip strength and muscle mass. However, whether household solid fuel use results in sarcopenia over time is not well elaborated.

**Methods:**

This study employed data from the 2011–2015 China Health and Retirement Longitudinal Study (CHARLS) that recruited 4,932 participants ≥45 years. The Cox proportional hazards regression model was conducted to estimate the impact of household solid fuel use for cooking and heating on sarcopenia development. The analysis was further stratified based on geographic position. Mediation analysis was employed to estimate the potential mediating effects of cognitive function and depressive symptoms associated with household solid fuel use and sarcopenia.

**Results:**

Over the 4-year follow-up, 476 cases of sarcopenia were reported (9.65%), with 254 in males (10.82%) and 222 in females (8.59%). Cooking and heating with solid fuels increased the risk of sarcopenia (Cooking: HR 1.401, 95% CI 1.138–1.724; Heating: HR 1.278, 95% CI 1.040–1.571). Crop residue/wood burning correlated with higher sarcopenia risk (Cooking: 1.420, 95% CI 1.147–1.758; Heating: 1.318, 95% CI 1.062–1.635). Switching to clean cooking fuels significantly reduced sarcopenia risk (HR 0.766, 95% CI 0.599–0.979). Heating with solid fuels was associated with higher sarcopenia risk only in southern China (HR 1.375, 95% CI 1.102–1.715). Additionally, cognitive function and depressive symptoms partially mediated the link between household solid fuel use and sarcopenia.

**Conclusion:**

Household use of solid fuels is associated with an increased risk of sarcopenia. Restricting the use of solid fuels and focusing on cognitive function and depressive symptoms in solid fuel users can help decrease sarcopenia development.

## 1 Introduction

Sarcopenia is a degenerative condition associated with aging in which skeletal muscle is progressively reduced in size, strength, and function (1, 2). As the global population ages, sarcopenia has become an increasingly pressing public health problem, especially in developing countries (3, 4). The approximate worldwide prevalence of sarcopenia among individuals over the age of 60 is 10% (5). Research has shown that skeletal muscle mass decreases by roughly 8% per decade beginning at the age of 40, with an accelerated decline observed among older individuals. After the age of 70, the decline rate further increases to 10–15% per decade (6). Notably, sarcopenia significantly increases the risk of adverse health consequences including falls, functional deterioration, frailty, and even mortality (1). Therefore, identifying contributing factors and implementing effective preventive measures for sarcopenia are vital concerns in public health.

The underlying mechanisms of sarcopenia are multifaceted, and in addition to established risk factors such as malnutrition, sedentary lifestyle, chronic diseases, and iatrogenic factors (1), recent studies have also revealed correlations between air pollutants and alterations in sarcopenia components (7–10). Previous evidence has demonstrated that exposure to air pollution can induce oxidative stress and inflammation (11), both of which have been identified as contributing factors to sarcopenia (12, 13). Indoor air pollution, resulting mainly from the burning of solid fuels in homes, is identified as one of the top 10 major risk factors for the global disease burden (14, 15). The burning of household solid fuels can generate harmful pollutants like particulate matter, nitrogen oxide, and sulfur oxides (16–18). Nearly 2.4 billion individuals depend on solid fuels to cook and/or heat, and indoor air pollution causes an estimated 3.2 million premature deaths annually (19). In a survey of 512,891 adults in 10 regions of China, a majority (52.1%) indicated the utilization of solid fuels for cooking or heating purposes, especially among rural residents (20). Numerous studies have demonstrated an association between exposure to indoor air pollution from the combustion of solid fuels and an increased risk of various health issues including arthritis (21), ischemic heart disease (22), depression (23), and cognitive decline (24, 25).

People spend the vast majority of their time indoors (88.9%), most of which is spent at home (26). Older adults tend to spend more time indoors (27), and their decline in overall physical health makes them more vulnerable to chronic health conditions and the negative effects of indoor air pollution (28). China, with the largest population of older adults (29), is experiencing an increasing burden of sarcopenia due to the rapidly aging demographic. To date, there is a lack of comprehensive longitudinal studies investigating the relationship between household solid fuel use and sarcopenia. Existing studies have ignored the large differences in fuel use between northern and southern China, as well as the potential influence of cognitive and psychological changes on the onset of sarcopenia.

In this study, we utilized a follow-up survey that included a nationally representative sample of Chinese adults aged 45 and above. The aim was to explore the association between household solid fuel use and sarcopenia, and to analyze the potential mediating effects of cognitive function and depressive symptoms within this association. The findings from this research will provide valuable evidence on the factors contributing to sarcopenia and support policy initiatives aimed at promoting the transition from inefficient and polluting solid fuels to cleaner alternatives. Additionally, addressing modifiable risk factors like cognitive decline and mental health concerns could help mitigate the burden of sarcopenia.

## 2 Methods

### 2.1 Data and sample

This study utilized follow-up data obtained from the China Health and Retirement Longitudinal Study (CHARLS). The CHARLS is a high-quality, nationally representative survey of Chinese individuals aged 45 and above. The individuals were recruited using a multistage probability sampling method in 28 provinces across China (30). After the 2011 baseline survey, follow-up surveys were performed every 2–3 years. Informed written consent was obtained from all subjects before participation, and the study gained ethical approval (approval numbers: IRB00001052-11015).

**Figure 1:**
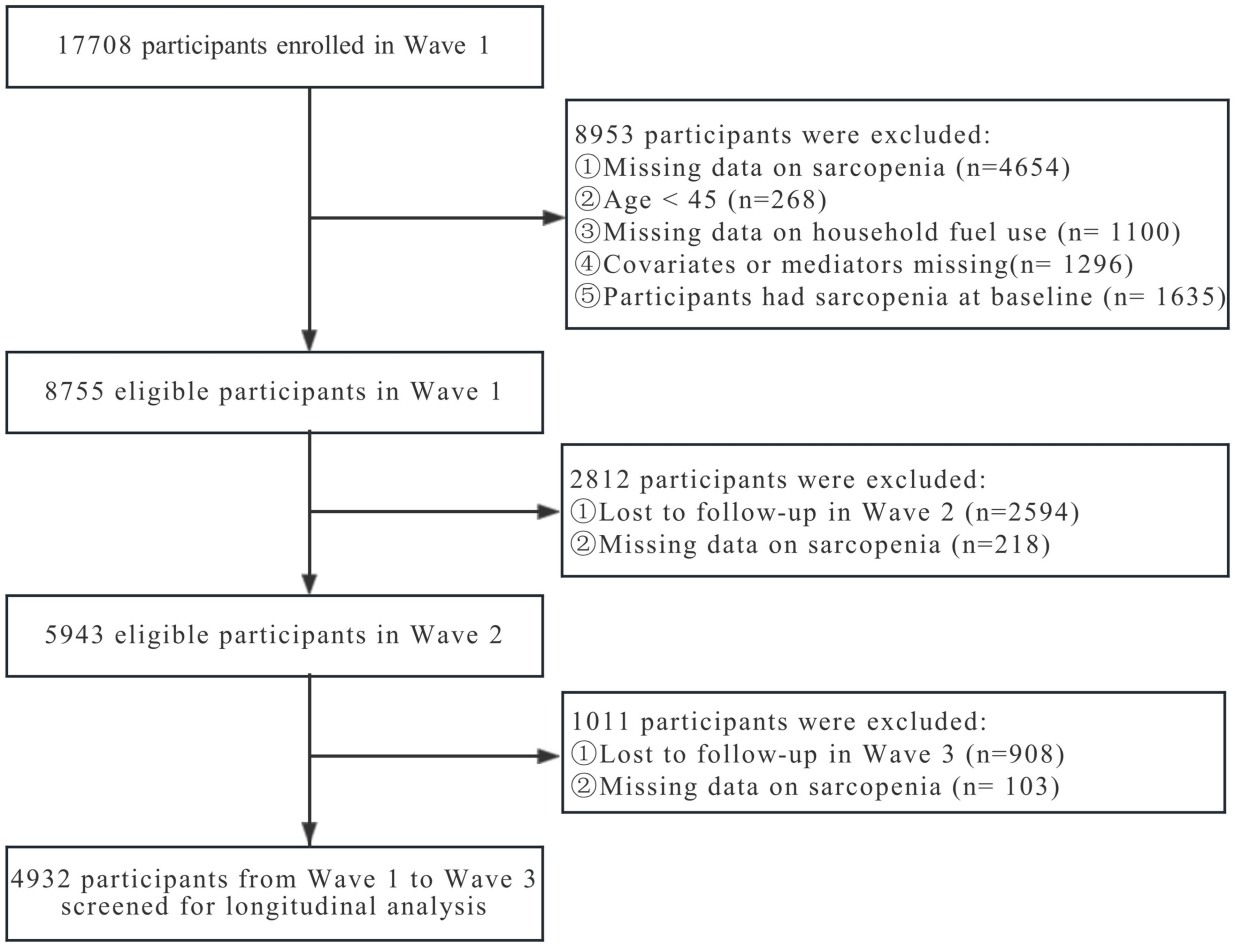
Flowchart of sample screened in the study.

There were 17,708 participants successfully enrolled in 2011. As information about physical examination was not provided in 2018, the first three waves (from 2011 to 2015) were selected for our study. We excluded 4,654 individuals due to missing information on sarcopenia at baseline. Our sample was restricted to adults aged 45 years and older, with 268 participants excluded for not meeting the age criteria. We also excluded observations with missing values on household fuel use (*n* = 1,100), and covariates or mediators (*n* = 1,296). Additionally, we excluded participants with sarcopenia at baseline (*n* = 1,635) or incomplete sarcopenia information in the follow-up surveys (*n* = 321), as well as those lost to follow-up (*n* = 3,522). Finally, our longitudinal study enrolled a total of 4,932 participants. Figure 1 provides an illustrated depiction of the comprehensive selection process followed for enrolling these participants.

### 2.2 Assessment of household solid fuel use

Household solid fuel use for cooking and heating was collected through a self-reported survey. Respondents were required to report their main fuel source for cooking and heating. Solid fuels were classified as crop residue/wood burning or coal, while clean fuels encompassed natural gas, marsh gas, liquefied petroleum gas, electricity, solar energy, or other sources (21, 23). Notably, we restricted our assessment of the impact of fuel switching on sarcopenia risk to cooking fuels data only, due to the substantial amount of missing data on heating fuels in 2015. Fuel switching was defined as participants who transitioned from one fuel type to another. For instance, those who utilized solid fuels for cooking at baseline but transitioned to clean fuels during follow-up were classified as having switched from solid fuel to clean fuel.

### 2.3 Assessment of sarcopenia

The assessment of sarcopenia followed the criteria outlined by the 2019 Asian Working Group for Sarcopenia (AWGS 2019) (31). The criteria involved evaluating three key factors: muscle strength, muscle mass, and physical performance. Sarcopenia is determined when there is a combination of low muscle mass along with either low muscle strength or low physical performance.

#### 2.3.1 Muscle strength

The study utilized grip strength measurements for determining overall muscle strength. Each participant’s maximum values for both left and right hands were tested twice, and the resulting values were averaged. In cases where a hand could not measure grip strength due to health reasons, the measurement from the other hand was considered. Insufficient grip strength was defined as <18 kg in females and < 28 kg in males (31).

#### 2.3.2 Muscle mass

The muscle mass was evaluated from appendicular skeletal muscle mass (ASM) using an anthropometric equation (32):


$$\begin{array}{l} ASM=0.193\times \mathrm{body weight}+0.107\times \mathrm{height}\\ \;-4.157\times \mathrm{gender}-0.037\times \mathrm{age}-2.631 \end{array}$$

In this equation, body weight was measured in kilograms, height in centimeters, age in years, and gender was coded as 1 for male and 2 for female. ASM/Ht^2^, which represents muscle mass adjusted for height, was obtained by dividing ASM by the square of height measured in centimeters. Low muscle mass was determined based on previous studies as ASM/Ht^2^ values below the 20th percentile (33, 34). In the study, the thresholds indicating low muscle mass were < 5.29 kg/m^2^ for females and < 7.01 kg/m^2^ for males.

#### 2.3.3 Physical performance

The physical performance of the participants was evaluated using the gait speed test and the five-time chair stand test. The gait speed test measured the participants’ normal walking speed (m/s) over a 2.5-m distance, performed both forward and backward. The analysis was conducted using the average of the two records. The five-time chair stand test evaluated the participants’ ability to stand and sit five consecutive times, with arms folded. Participants unable to complete the test were classified as having low physical performance. Low physical performance was determined as a gait speed less than 1.0 m/s or a five-time stand chair test lasting 12 s or longer (31).

### 2.4 Covariates and mediators

Based on previous studies, we selected potential covariates associated with household solid fuel use and sarcopenia, which have been categorized into two groups: sociodemographic variables and health-related factors. Sociodemographic variables included age (<60 or ≥ 60), gender (male or female), marital status (married/cohabited or others), residential area (rural or urban), geographic position (north or south), education level (illiterate, primary school or below, middle school, or high school or above), and economic situation (good, fair, or poor). Health-related factors included smoking status (no or yes), drinking status (no or yes), and number of chronic diseases (0, 1, or ≥ 2). Additionally, we included baseline cognitive function and depressive symptoms as possible mediators in the mediation analysis.

In our study, the geographic position was determined using the Qinling-Huaihe line in China (35). The self-reported family economic situation was categorized into three groups: “very high” and “relatively high” were grouped as “good,” “average” was classified as “fair,” and “relatively poor” and “poor” were grouped as “poor” (36). The assessment of cognitive function involved measuring intelligence and episodic memory using a scoring system ranging from 0 to 31. A higher score on this assessment demonstrates better cognitive function (24, 25). The score for the assessment of depressive symptoms ranges from 0 to 30, with higher scores reflecting a greater presence of depressive symptoms (23).

### 2.5 Statistical analysis

In this study, means ± standard deviations (SDs) were used to present continuous variables, while categorical variables were reported as numbers (percentages). To analyze the differences in characteristics between different types of household solid fuel used for cooking and heating at baseline, *t*-tests were conducted for continuous data, and Chi-square tests were employed for categorical data.

To examine the association between household solid fuel use and sarcopenia, Cox proportional hazards regression models were utilized. We performed a stratified analysis based on geographic position, considering that northern China is classified as a central heating area during winter (37, 38). The results were presented as hazard ratios (HR) along with their corresponding 95% confidence intervals (CI). The endpoint of this study was the occurrence of sarcopenia, and the time scale used ranged from 0 to 4 (0, 2, and 4). Furthermore, we included cognitive function and depressive symptoms as potential mediators in the mediation analyses. These analyses were conducted using functions implemented in the R package “mediation” and employed the nonparametric bootstrap method simulation approach with 5,000 iterations.

The following variables were adjusted in all models to control for potential confounding factors: sociodemographic variables (age, gender, marital status, residential area, geographic position, education level, and economic situation) and health-related factors (smoking status, drinking status, and number of chronic diseases). All statistical analyses were performed utilizing Stata 17 and R 4.3.1 software. A significance level of *p* < 0.05 (two-tailed) was applied to determine statistical significance.

## 3 Results

### 3.1 Basic characteristics of the selected participants

There were 4,932 participants involved in this study, 52.41% of whom were females and 37.49% were aged 60 years or above. At baseline, 60.69 and 60.28% of individuals reported primary use of solid fuels for cooking and heating respectively, while 39.31 and 39.72% used clean fuels for cooking and heating, respectively. Participants who used solid fuels as their main energy for cooking/heating were more likely to live in rural regions and northern China, possess lower educational attainment, report worse economic status, do not smoke, and exhibit a higher burden of chronic disease. Additionally, those using solid cooking fuel attained lower scores on cognitive function and reported more depression symptoms. Additionally, we observed no significant differences in household fuel use types relative to gender, marital status, or drinking status (Table 1).

**Table 1:** Baseline characteristics of enrolled participants.

Characteristics	Cooking fuels		*p*	Heating fuels		*p*
	Clean	Solid		Clean	Solid	
*N*	1,939 (39.31)	2,993 (60.69)		1,959 (39.72)	2,973 (60.28)	
Age			0.001			0.268
<60	1,265 (65.24)	1,818 (60.74)		1,243 (63.45)	1,840 (61.89)	
≥60	674 (34.76)	1,175 (39.26)		716 (36.55)	1,133 (38.11)	
Gender			0.874			0.476
Male	920 (47.45)	1,427 (47.68)		920 (46.96)	1,427 (48.00)	
Female	1,019 (52.55)	1,566 (52.32)		1,039 (53.04)	1,546 (52.00)	
Marital status			0.177			0.007
Married/Cohabited	1,752 (90.36)	2,738 (91.48)		1,757 (89.69)	2,733 (91.93)	
Others	187 (9.64)	255 (8.52)		202 (10.31)	240 (8.07)	
Residential area			<0.001			<0.001
Rural	1,416 (73.03)	2,842 (94.95)		1,522 (77.69)	2,736 (92.03)	
Urban	523 (26.97)	151 (5.05)		437 (22.31)	237 (7.97)	
Geographic position			<0.001			<0.001
North	668 (34.45)	1,564 (52.26)		289 (14.75)	1,943 (65.35)	
South	1,271 (65.55)	1,429 (47.74)		1,670 (85.25)	1,030 (34.65)	
Education level			<0.001			0.006
Illiterate	352 (18.15)	878 (29.34)		449 (22.92)	781 (26.27)	
Primary school or below	843 (43.48)	1,318 (44.04)		853 (43.54)	1,308 (44.00)	
Middle school	481 (24.81)	597 (19.95)		448 (22.87)	630 (21.19)	
High school or above	263 (13.56)	200 (6.68)		209 (10.67)	254 (8.54)	
Economic situation			<0.001			0.003
Good	59 (3.04)	60 (2.00)		60 (3.06)	59 (1.98)	
Fair	1,123 (57.92)	1,567 (52.36)		1,101 (56.20)	1,589 (53.45)	
Poor	757 (39.04)	1,366 (45.64)		798 (40.74)	1,325 (44.57)	
Smoking status			0.033			0.003
No	1,223 (63.07)	1,797 (60.04)		1,249 (63.76)	1,771 (59.57)	
Yes	716 (36.93)	1,196 (39.96)		710 (36.24)	1,202 (40.43)	
Drinking status			0.193			0.823
No	1,274 (65.70)	2,020 (67.49)		1,312 (66.97)	1,982 (66.67)	
Yes	665 (34.30)	973 (32.51)		647 (33.03)	991 (33.33)	
Number of chronic diseases			0.005			0.012
0	672 (34.66)	983 (32.84)		660 (33.69)	995 (33.47)	
1	620 (31.98)	877 (29.30)		635 (32.41)	862 (28.99)	
≥2	647 (33.37)	1,133 (37.85)		664 (33.89)	1,116 (37.54)	
Cognitive function	15.52 ± 5.00	14.00 ± 5.04	<0.001	14.95 ± 5.15	14.36 ± 5.02	<0.001
Depressive symptoms	6.98 ± 5.54	8.85 ± 6.45	<0.001	14.95 ± 5.16	8.68 ± 6.35	<0.001
Handgrip strength (kg)	32.72 ± 9.58	31.98 ± 11.23	0.017	32.41 ± 9.31	32.18 ± 11.40	0.464
Gait speed (m/s)	0.80 ± 2.80	0.75 ± 2.38	0.692	0.79 ± 2.71	0.75 ± 2.42	0.751
5-time chair stand test (s)	9.86 ± 3.61	10.68 ± 3.86	<0.001	9.85 ± 3.56	10.70 ± 3.89	<0.001
ASM/Ht^2^ (kg/m^2^)	6.97 ± 1.02	6.90 ± 1.02	0.036	6.90 ± 1.03	6.95 ± 1.02	0.080

Values were means ± SD or *n* (percentages). Due to rounding, polytomous variable values may not add up to 100%. *p* values were calculated using analysis of *t*-test and Chi-square test for continuous and categorical variables, respectively.

### 3.2 Association between household solid fuel use and sarcopenia

There were 297 (6.02%) new occurrences of sarcopenia in 2013, 179 (3.63%) in 2015, and a total of 476 (9.65%) incident cases of sarcopenia overall, with 254 (10.82%) occurring in males and 222 (8.59%) in females. Moreover, individuals who developed sarcopenia tended to be older, male, reside in southern regions or rural areas, smoke, exhibit a lower education level, lower cognitive function, and more depressive symptoms (Supplementary Table S1). During the 19,134 person-years of follow-up, the incidence rate of sarcopenia was higher among individuals who utilized solid fuels for cooking or heating compared to those who used clean fuels (Cooking: 28.85 vs. 18.84 per 1,000 person-years; Heating: 24.97 vs. 24.73 per 1,000 person-years). In the unadjusted model (model 1), the occurrence of sarcopenia was associated with the primary use of solid fuels for cooking, whereas heating demonstrated no association. However, after sociodemographic and health-related factors were taken into account (model 3), both the consumption of solid fuels for cooking (HR: 1.401; 95% CI: 1.138–1.724) and heating (HR: 1.278; 95% CI: 1.040–1.571) were found to be linked to an increased risk of developing sarcopenia (Table 2; Supplementary Figure S2). Different types of solid fuel use can also impact the development of sarcopenia. Participants who used crop residues or wood for cooking (HR: 1.420; 95% CI: 1.147–1.758) and heating (HR: 1.318; 95% CI: 1.062–1.635) had a significantly higher risk of developing sarcopenia than those who used coal (Table 2).

**Table 2:** Incidence rates and hazard ratios with sarcopenia by household fuel types according to cooking and heating.

Exposure	Events	Incidence rate per 1,000 person-years		HR (95% CI)	
			Model 1	Model 2	Model 3
Cooking					
Household fuel use					
Clean fuel	143	18.84	Reference	Reference	Reference
Solid fuel	333	28.85	1.527 (1.255, 1.857)	1.400 (1.138, 1.722)	1.401 (1.138, 1.724)
Types of household fuel use					
Clean fuel	143	18.84	Reference	Reference	Reference
Coal	52	20.05	1.064 (0.774, 1.461)	1.303 (0.937, 1.812)	1.286 (0.925, 1.788)
Crop residue/Wood	281	31.40	1.660 (1.357, 2.031)	1.420 (1.147, 1.758)	1.425 (1.151, 1.765)
Heating					
Household fuel use					
Clean fuel	188	24.73	Reference	Reference	Reference
Solid fuel	288	24.97	1.010 (0.840, 1.213)	1.260 (1.026, 1.548)	1.278 (1.040, 1.571)
Types of household fuel use					
Clean fuel	188	24.73	Reference	Reference	Reference
Coal	111	17.79	0.720 (0.570, 0.911)	1.116 (0.840, 1.482)	1.133 (0.852, 1.505)
Crop residue/Wood	177	33.45	1.350 (1.099, 1.657)	1.318 (1.062, 1.635)	1.336 (1.076, 1.659)
Transition of cooking fuel					
Persistent clean fuel	114	17.77	Reference	Reference	Reference
Change from clean to solid	29	24.62	1.380 (0.920, 2.080)	1.414 (0.933, 2.143)	1.367 (0.900, 2.075)
Persistent solid fuel	243	30.98	Reference	Reference	Reference
Change from solid to clean	90	24.34	0.790 (0.620, 1.000)	0.772 (0.604, 0.987)	0.766 (0.599, 0.979)

HR, Hazard ratio; CI, Confidence interval. Model 1 was conducted without any adjustment; Model 2 was adjusted for the sociodemographic variables (age, gender, marital status, residential area, geographic position, educational level, and economic situation); Model 3 was further adjusted for health-related factors (smoking status, drinking status, and number of chronic diseases).

Among the participants surveyed at baseline, 1,939 used clean fuels and 2,993 used solid fuels to cook. In the subsequent follow-up, 303 individuals transitioned from clean to solid fuels, whereas 955 participants made the switch from solid to clean fuels (Supplementary Table S2). Participants with a persistent reliance on solid fuels for cooking displayed the highest incidence rate of sarcopenia, recording a rate of 30.98 per 1,000 person-years. Conversely, participants who consistently used clean fuels exhibited the lowest incidence rate at 17.77 per 1,000 person-years. After adjustment for all covariates, the final model (Model 3) revealed that transitioning from the persistent use of solid fuels for cooking to clean fuels decreased the risk of sarcopenia (HR: 0.766; 95% CI: 0.599–0.979). Conversely, transitioning from the persistent use of clean fuels to solid fuels potentially increased the risk of sarcopenia (HR: 1.367; 95% CI: 0.900–2.075), despite no statistical significance (Table 2).

### 3.3 Stratified analyses between household solid fuel use and sarcopenia

We performed stratified analyses based on geographic position. After adjusting for all covariates, our findings revealed significant associations between cooking with solid fuels and a higher risk of sarcopenia, irrespective of whether in the northern (HR: 1.646; 95% CI: 1.042–2.601) or southern (HR: 1.348; 95% CI: 1.065–1.707) regions. Notably, in the southern region, the use of solid fuels for heating was significantly associated with a higher risk of sarcopenia (HR: 1.375; 95% CI: 1.102–1.715), whereas no significant difference was observed in northern China (HR: 0.900; 95% CI: 0.537–1.506) (Table 3).

**Table 3:** Hazard ratios with sarcopenia by household fuel exposure stratified by geographic position at baseline.

Model	Geographic position	Cooking with solid fuel^a^		Heating with solid fuel^b^	
		HR (95% CI)	*p*	HR (95% CI)	*p*
Model 1	North	2.100 (1.353, 3.257)	0.001	1.088 (0.656, 1.807)	0.743
	South	1.647 (1.317, 2.060)	<0.001	1.578 (1.274, 1.955)	<0.001
Model 2	North	1.649 (1.044, 2.605)	0.032	0.892 (0.533, 1.490)	0.662
	South	1.340 (1.059, 1.696)	0.015	1.353 (1.085, 1.687)	0.007
Model 3	North	1.646 (1.042, 2.601)	0.033	0.900 (0.537, 1.506)	0.688
	South	1.348 (1.065, 1.707)	0.013	1.375 (1.102, 1.715)	0.005

a Reference group was cooking with clean fuel.

b Reference group was heating with clean fuel.

HR, Hazard ratio; CI, Confidence interval. Model 1 was performed without any adjustment; Model 2 was adjusted for the sociodemographic variables (age, gender, marital status, residential area, educational level, and economic situation); Model 3 was further adjusted for health-related factors (smoking status, drinking status, and number of chronic diseases).

### 3.4 Mediation analyses between household solid fuel use and sarcopenia

**Figure 2:**
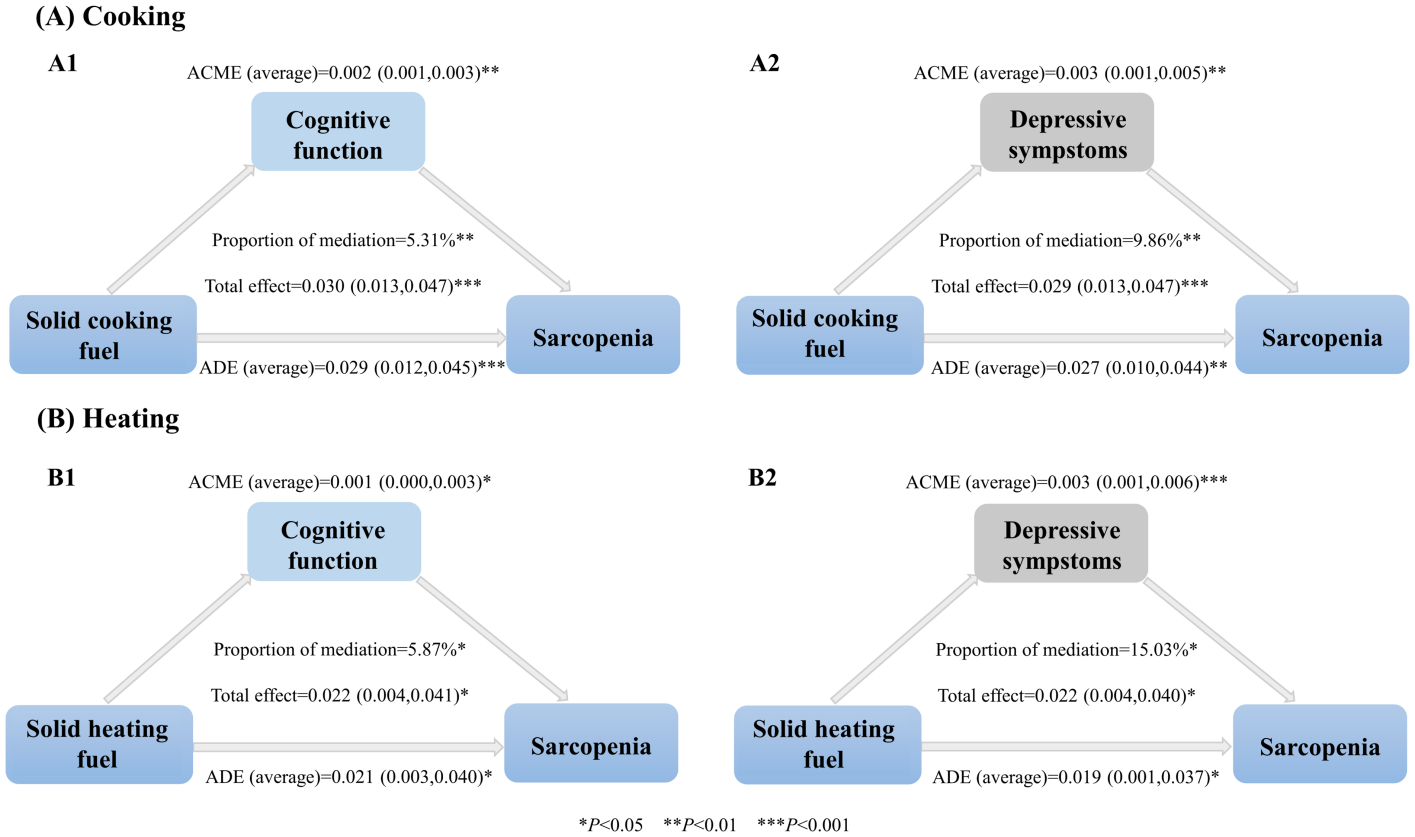
Association between household solid fuel use, mediation variables, and sarcopenia. ACME, The average causal mediation effect; ADE, The average direct effect. Proportion of mediation = ACME (average)/Total effect. ACME represents the effect of household solid fuel use on sarcopenia through mediators. ADE refers to the direct effect of household solid fuel use on sarcopenia. All mediation models were adjusted by sociodemographic variables (age, gender, marital status, residential area, geographic position, educational level, and economic situation) and health-related factors (smoking status, drinking status, and number of chronic diseases).

In the mediation analyses, it was found that cognitive function and depressive symptoms played a partial mediating role in the association between household solid fuel use (including cooking and heating) and sarcopenia. Specifically, cognitive function mediated 5.31% (*p* = 0.002) of the effect of cooking solid fuels on sarcopenia and 5.87% (*p* = 0.031) of the effect of heating solid fuels on sarcopenia. Depressive symptoms, on the other hand, had a greater proportion of the mediation effects. For cooking solid fuels, depressive symptoms mediated 9.86% (*p* = 0.003) of the effect on sarcopenia, and for heating solid fuels, depressive symptoms mediated 15.03% (*p* = 0.020) of the effect on sarcopenia (Figure 2).

## 4 Discussion

In this population-based cohort study of Chinese individuals aged over 45 years, we identified a significant association between the use of solid fuels in households and an elevated risk of sarcopenia. Notably, the transition from solid fuels to clean fuels for cooking demonstrated a substantial impact on reducing the incidence rate of sarcopenia. Furthermore, the association between sarcopenia and household solid fuel use for heating was observed exclusively in southern China, likely due to regional variations in heating patterns. Additionally, cognitive function and depressive symptoms partially mediated the relationship between household solid fuel use and sarcopenia. These findings offer a unique environmental perspective that can inform public health authorities in their efforts to prevent and manage sarcopenia.

One important finding of our study indicated that the use of solid fuels for household cooking and heating increases the risk of sarcopenia, with potentially greater severity observed in the context of cooking with solid fuels. Our study is in accordance with these previous studies that have also highlighted the association between physical health issues and indoor air pollution (15, 21, 22, 36). Existing research has also suggested that exposure to ambient air pollution may contribute to a higher risk of sarcopenia and its associated components (7, 10, 39). The patterns and frequencies of solid fuel exposure differ significantly when households are engaged in cooking and heating activities (40, 41). Cooking represents an integral part of daily human life, leading to lifelong exposure, whereas combustion of solid fuels for heating occurs only in specific circumstances, such as cold winters. Therefore, the regular use of solid fuels for cooking results in prolonged exposure to indoor pollution for household members, thereby increasing the susceptibility to sarcopenia. Over a 4-year follow-up period, we analyzed the occurrence of sarcopenia among participants who switched cooking fuels. The results revealed a significant reduction in the risk of sarcopenia when transitioning from solid to clean fuels. This finding is consistent with similar studies (25, 42). Conversely, transitioning from clean to solid fuels appears to increase the risk of sarcopenia, although there is no statistical significance. Possible reasons include the limited sample size of participants switching from clean to solid fuels and the fact that the impact of air pollution on the body is a chronic and progressive process that manifests long-term health effects (43), a 4-year follow-up period may not be enough to observe a sufficient number of outcome events. In China, the Qinling-Huaihe Line divides the country into northern and southern regions (35, 37), the longer and colder winters in the northern region have led to the widespread adoption of centralized heating patterns, while traditional heating methods are still predominantly used in the southern region (37, 38). For most households in the northern region, indoor air pollution resulting from heating with solid fuels appears to be comparatively lower compared to the southern region, which potentially elucidates the association between the use of solid fuels for heating and the risk of sarcopenia that exists only in southern China. Our study findings highlight the effectiveness of promoting the use of clean fuels and adopting scientifically sound heating methods as an approach to reduce the occurrence of sarcopenia.

Another significant result from our analysis was the partial mediating effect of cognitive function and depressive symptoms in the association between household solid fuel use and sarcopenia. To our knowledge, few studies have examined the mediating role of cognitive functioning and depressive symptoms between household solid fuel use and sarcopenia. Previous research has shown that the burning of solid fuels is linked to decreased cognitive function (25) and increased depressive symptoms (23). A meta-analysis has identified depression and cognitive impairment as significant risk factors for sarcopenia (44). Depression may contribute to the occurrence of sarcopenia through reduced physical activity, upregulation of inflammatory cytokines, and dysregulation of the hypothalamic–pituitary–adrenal axis (45, 46). Sarcopenia and cognitive decline share common pathophysiological pathways, including oxidative stress and chronic inflammation (47). Impaired cognitive function can lead to decreased physical activity and reduced dietary intake, thereby accelerating the development of sarcopenia (33). Furthermore, a study conducted in China suggested that cognitive decline could serve as an early sensitive marker for gait speed, with poor baseline cognitive function predicting subsequent declines in gait speed after 4 years (48). Our findings present a novel perspective for preventing the occurrence of sarcopenia among long-term household solid fuel users by focusing on modifiable risk factors such as cognitive function and mental health, and implementing proactive measures to reduce the risk of sarcopenia to some extent. Nevertheless, the relationship between sarcopenia and depressive symptoms remains uncertain and may be bidirectional. Some studies suggest that sarcopenia may lead to depression (49, 50), while others propose that depression contributes to sarcopenia through behavioral and physiological changes (44, 45). Therefore, further studies are needed to clarify the causal pathways linking sarcopenia, cognitive function, and depressive symptoms, particularly in the context of long-term solid fuel use. Elucidating these mechanisms will be critical for developing effective interventions to mitigate the risk of sarcopenia.

The precise biological mechanisms underlying the link between indoor air pollution from solid fuel use and sarcopenia remain inadequately understood. Existing studies provide some clues: (1) Air pollution has the potential to induce oxidative stress and mitochondrial dysfunction (11, 51), which can subsequently affect muscle function (4, 13); (2) Exposure to different air pollutants increases the production of pro-inflammatory mediators (52), potentially leading to muscle loss, decreased muscle mass, and reduced strength (12, 53); (3) Exposure to air pollution has been associated with modifications in DNA methylation patterns across an individual’s lifespan (54). These alterations in the epigenetic regulation of genes have the potential to contribute to the development of impaired muscle function during later stages of life (55); and (4) Exposure to air pollution has been linked to a higher risk of insulin resistance (56), which is considered a contributing factor to the development of sarcopenia (13).

Cooking and heating with solid fuels are widely acknowledged as the primary contributors to indoor air pollution in developing countries. In this study, we found that participants who used solid fuels for cooking/heating were more prone to live in rural and northern China, and were associated with lower levels of education and poorer economic conditions. Previous studies have indicated that low income influences household fuel choices, with higher-income individuals being more inclined toward cleaner fuel options, while lower-income individuals are more likely to rely on solid fuels for cooking and heating purposes (57, 58). Furthermore, fuel choices differ due to various factors, including household size and composition, cooking and heating practices, geographical location, and the level of urbanization (57). Our study also found that for different types of solid fuels, burning traditional biomass fuels (e.g., crop residue/wood) was more harmful to sarcopenia than other fuels. This indicates the need for solid fuel users to switch from solid fuels to cleaner fuels. The utilization of polluting fuels necessitates extensive time investment in fuel collection and preparation, as well as the use of inefficient cooking devices. Without proactive policy actions, it is anticipated that by the year 2030, around 2.1 billion individuals will continue to lack access to clean fuels and technologies (19). Similarly, China faces a substantial public health risk due to the pollution caused by indoor solid fuel usage. Solid fuels are commonly used for cooking and heating purposes in rural Chinese households, particularly in western provinces such as Tibet, Qinghai, and Ningxia, where approximately 95% of rural families depend on traditional solid fuels for heating (59). Given these considerations, it is crucial to prioritize actions aimed at reducing the dependence on solid fuels, enhancing energy infrastructure, expanding access to clean energy sources, and implementing effective renewable energy policies. This is especially critical in economically underdeveloped rural areas.

This study makes several notable strengths in this field. Firstly, it utilizes a nationally representative prospective cohort dataset in China, enhancing the findings’ reliability and generalizability. Secondly, multiple measurements of cooking fuel types were conducted to evaluate the effects of fuel transition on the risk of sarcopenia. Thirdly, the study compares the disparities in sarcopenia risk associated with cooking and heating fuel usage between the northern and southern regions of China. Furthermore, potential mediating factors were assessed, further supporting research on the underlying mechanisms. These findings provide robust evidence that supports the promotion of clean fuel policies. The findings underscore the significance of implementing clean fuel policies in safeguarding public health and reducing the prevalence of sarcopenia. However, this study has some limitations: (1) Rather than directly measuring indoor environmental pollutant concentrations, we relied on proxy variables by using self-reported primary fuel types for cooking and heating. This approach may introduce measurement errors and imprecision. (2) Due to the limitations of the CHARLS, recommended methods, such as dual-energy X-ray absorptiometry (DXA) or bioelectrical impedance analysis (BIA) (31), were not employed for estimating muscle mass. Nevertheless, a validated anthropometric equation specific to the Chinese population was utilized (32). (3) The study period was limited to 4 years, which may restrict the comprehensive assessment of alterations in muscle mass and function attributed to the use of household solid fuel. (4) Despite considering a relatively extensive range of confounding factors, residual confounding from variables like protein intake and physical activity may still have an impact due to insufficient or missing data in CHARLS. Future studies should employ longer-term and more comprehensive cohort designs to elucidate the mechanistic role of household consumption of solid fuel in the development of sarcopenia. Furthermore, there is an urgent imperative to conduct direct quantitative analyses to explore the association between various types of indoor air pollutants and the occurrence of sarcopenia.

## 5 Conclusion

In conclusion, our findings indicate an association between the use of household solid fuel and an increased risk of sarcopenia. Cognitive function and depressive symptoms may partially mediate this association. This suggests that mitigating the burden of sarcopenia can be achieved through modifiable risk factors, including the prevention of cognitive decline and addressing mental health concerns. Switching fuels from solid to clean has the potential to reduce the incidence of sarcopenia, underscoring the importance of promoting and disseminating the use of clean household fuels worldwide to promote health. Moving forward, research aimed at developing evidence-based policy recommendations to facilitate the adoption of clean fuels will be vital in reducing the health risks associated with household solid fuel use. Given the widespread use of solid fuels, our study makes substantial contributions to informing future policy and program development in the field of public health. These endeavors possess the potential to alleviate the detrimental consequences of indoor air pollution, improve physical and mental well-being, and mitigate associated health burdens both domestically and globally.

## Data Availability

Publicly available datasets were analyzed in this study. This data can be found at: http://charls.pku.edu.cn/en.
