# Supplementary material for: Effects of household solid fuel use on sarcopenia in middle-aged and older adults: evidence from a nationwide cohort study
Source: Front Public Health. 2024 Oct 2;12:1337979. doi: 10.3389/fpubh.2024.1337979 (PMC11479963; doi:10.3389/fpubh.2024.1337979)
Supplement: Supplementary file 1 [file Data_Sheet_1.docx]

**Effects of household solid fuel use on sarcopenia in middle-aged and older adults: Evidence from a nationwide cohort study**

Supplementary Material

**Table S1** Baseline characteristics according to sarcopenia status.

**Table S2** Baseline characteristics of participants that switched their cooking fuels during follow-up.

**Figure S1** The spatial distribution of 4932 participants in the analysis from different provinces in China.

**Figure S2** Forest plot of multifactorial Cox analysis between household solid fuel use and sarcopenia.

| **Table S1** Baseline characteristics according to sarcopenia status. | | | | |
| --- | --- | --- | --- | --- |
| **Characteristics** | **Total** | **Non-sarcopenia** | **Sarcopenia** | ***P*** |
| **N** | 4932 | 4456 (90.35) | 476 (9.65) |  |
| **Age** |  |  |  | <0.001 |
| <60 | 3083 (62.51) | 2876 (64.54) | 207 (43.49) |  |
| ≥60 | 1849 (37.49) | 1580 (35.46) | 269 (56.51) |  |
| **Gender** |  |  |  | 0.008 |
| Male | 2347 (47.59) | 2093 (46.97) | 254 (53.36) |  |
| Female | 2585 (52.41) | 2363 (53.03) | 222 (46.64) |  |
| **Marital status** |  |  |  | <0.001 |
| Married/Cohabited | 4490 (91.04) | 4082 (91.61) | 408 (85.71) |  |
| Others | 442 (8.96) | 374 (8.39) | 68 (14.29) |  |
| **Residential area** |  |  |  | <0.001 |
| Rural | 4258 (86.33) | 3817 (85.66) | 441 (92.65) |  |
| Urban | 674 (13.67) | 639 (14.34) | 35 (7.35) |  |
| **Geographic position** |  |  |  | <0.001 |
| North | 2232 (45.26) | 2091 (46.93) | 141 (29.62) |  |
| South | 2700 (54.74) | 2365 (53.07) | 335 (70.38) |  |
| **Education level** |  |  |  | <0.001 |
| Illiterate | 1230 (24.94) | 1079 (24.21) | 151 (31.72) |  |
| Primary school or below | 2161 (43.82) | 1913 (42.93) | 248 (52.1) |  |
| Middle school | 1078 (21.86) | 1019 (22.87) | 59 (12.39) |  |
| High school or above | 463 (9.39) | 445 (9.99) | 18 (3.78) |  |
| **Economic situation** |  |  |  | 0.083 |
| Good | 119 (2.41) | 114 (2.56) | 5 (1.05) |  |
| Fair | 2690 (54.54) | 2436 (54.67) | 254 (53.36) |  |
| Poor | 2123 (43.05) | 1906 (42.77) | 217 (45.59) |  |
| **Smoking status** |  |  |  | <0.001 |
| No | 3020 (61.23) | 2773 (62.23) | 247 (51.89) |  |
| Yes | 1912 (38.77) | 1683 (37.77) | 229 (48.11) |  |
| **Drinking status** |  |  |  | 0.064 |
| No | 3294 (66.79) | 2958 (66.38) | 336 (70.59) |  |
| Yes | 1638 (33.21) | 1498 (33.62) | 140 (29.41) |  |
| **Number of chronic diseases** |  |  |  | 0.058 |
| 0 | 1655 (33.56) | 1472 (33.03) | 183 (38.45) |  |
| 1 | 1497 (30.35) | 1365 (30.63) | 132 (27.73) |  |
| ≥2 | 1780 (36.09) | 1619 (36.33) | 161 (33.82) |  |
| **Cognitive function** | 14.60±5.08 | 14.77±5.03 | 12.98±5.26 | <0.001 |
| **Depressive symptoms** | 8.11±6.18 | 7.98±6.10 | 9.35±6.77 | <0.001 |

Values were means ± SD or n (percentages). Due to rounding, polytomous variable values may not add up to 100%. *P* values were calculated using analysis of *t*-test and Chi-square test for continuous and categorical variables, respectively.

| **Table S2** Baseline characteristics of participants that switched their cooking fuels during follow-up. | | | | | |
| --- | --- | --- | --- | --- | --- |
| **Characteristics** | **Always clean** | **Solid to clean** | **Clean to solid** | **Always solid** | ***P*** |
| **N** | 1636 (33.17) | 955 (19.36) | 303 (6.14) | 2038 (41.32) |  |
| **Age** |  |  |  |  | 0.001 |
| <60 | 1072 (65.53) | 609 (63.77) | 193 (63.70) | 1209 (59.32) |  |
| >=60 | 564 (34.47) | 346 (36.23) | 110 (36.30) | 829 (40.68) |  |
| **Gender** |  |  |  |  | 0.771 |
| Male | 773 (47.25) | 443 (46.39) | 147 (48.51) | 984 (48.28) |  |
| Female | 863 (52.75) | 512 (53.61) | 156 (51.49) | 1054 (51.72) |  |
| **Marital status** |  |  |  |  | 0.064 |
| Married/Cohabited | 1476 (90.22) | 857 (89.74) | 276 (91.09) | 1881 (92.30) |  |
| Others | 160 (9.78) | 98 (10.26) | 27 (8.91) | 157 (7.70) |  |
| **Residential area** |  |  |  |  | <0.001 |
| Rural | 1153 (70.48) | 880 (92.15) | 263 (86.80) | 1962 (96.27) |  |
| Urban | 483 (29.52) | 75 (7.85) | 40 (13.20) | 76 (3.73) |  |
| **Geographic position** |  |  |  |  | <0.001 |
| North | 519 (31.72) | 408 (42.72) | 149 (49.17) | 1156 (56.72) |  |
| South | 1117 (68.28) | 547 (57.28) | 154 (50.83) | 882 (43.28) |  |
| **Education level** |  |  |  |  | <0.001 |
| Illiterate | 275 (16.81) | 232 (24.29) | 77 (25.41) | 646 (31.70) |  |
| Primary school or below | 718 (43.89) | 427 (44.71) | 125 (41.25) | 891 (43.72) |  |
| Middle school | 409 (25.00) | 226 (23.66) | 72 (23.76) | 371 (18.20) |  |
| High school or above | 234 (14.30) | 70 (7.33) | 29 (9.57) | 130 (6.38) |  |
| **Economic situation** |  |  |  |  | <0.001 |
| Good | 50 (3.06) | 18 (1.88) | 9 (2.97) | 42 (2.06) |  |
| Fair | 947 (57.89) | 506 (52.98) | 176 (58.09) | 1061 (52.06) |  |
| Poor | 639 (39.06) | 431 (45.13) | 118 (38.94) | 935 (45.88) |  |
| **Smoking status** |  |  |  |  | 0.054 |
| No | 1043 (63.75) | 586 (61.36) | 180 (59.41) | 1211 (59.42) |  |
| Yes | 593 (36.25) | 369 (38.64) | 123 (40.59) | 827 (40.58) |  |
| **Drinking status** |  |  |  |  | 0.194 |
| No | 1062 (64.91) | 648 (67.85) | 212 (69.97) | 1372 (67.32) |  |
| Yes | 574 (35.09) | 307 (32.15) | 91 (30.03) | 666 (32.68) |  |
| **Number of chronic diseases** |  |  |  |  | 0.022 |
| 0 | 566 (34.60) | 323 (33.82) | 106 (34.98) | 660 (32.38) |  |
| 1 | 536 (32.76) | 275 (28.80) | 84 (27.72) | 602 (29.54) |  |
| ≥2 | 534 (32.64) | 357 (37.38) | 113 (37.29) | 776 (38.08) |  |

Values were n (percentages). Due to rounding, polytomous variable values may not add up to 100%. *P* values were calculated using the Chi-square test.

**
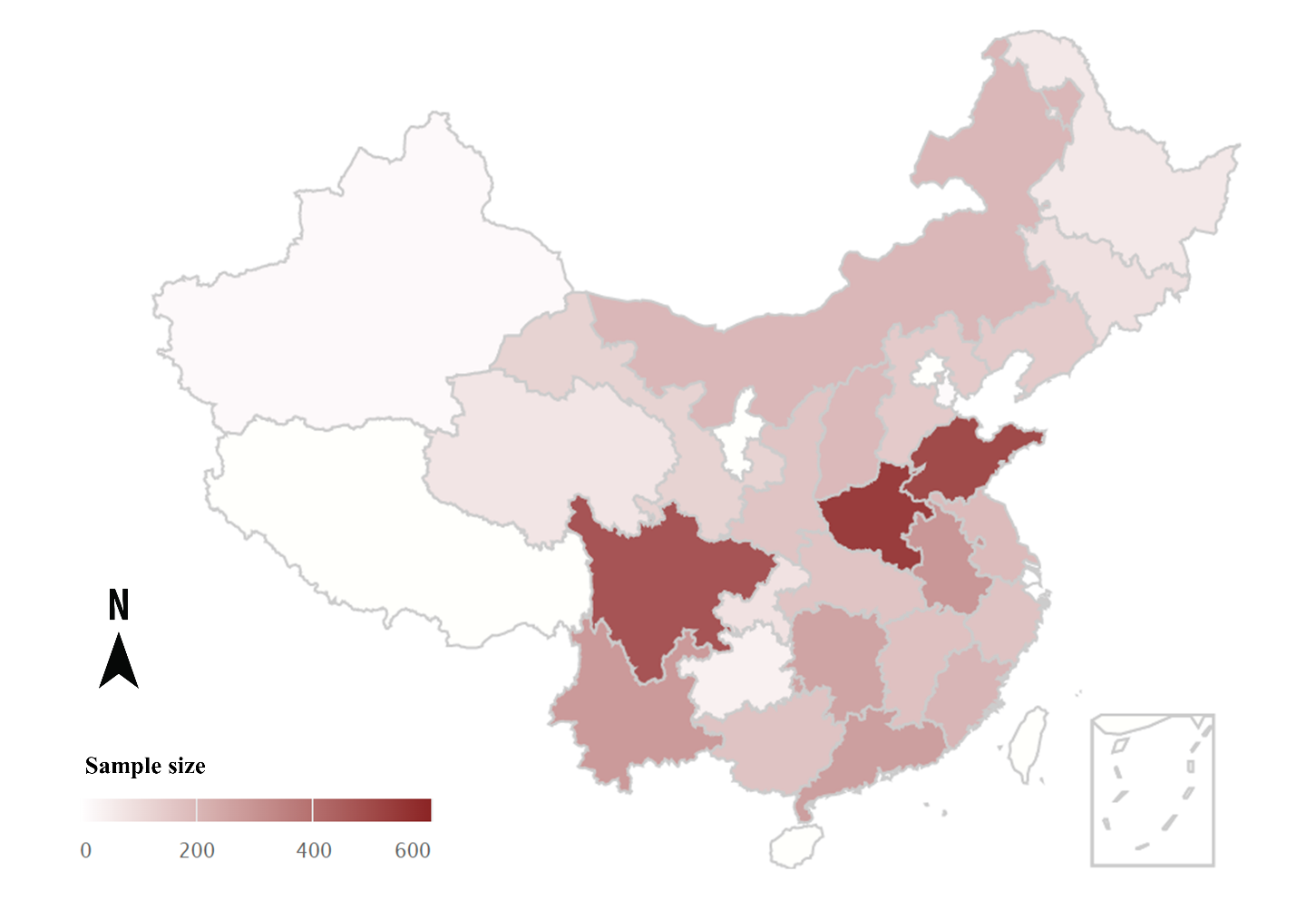
Figure S1** The spatial distribution of 4932 participants in the analysis from different provinces in China.

**
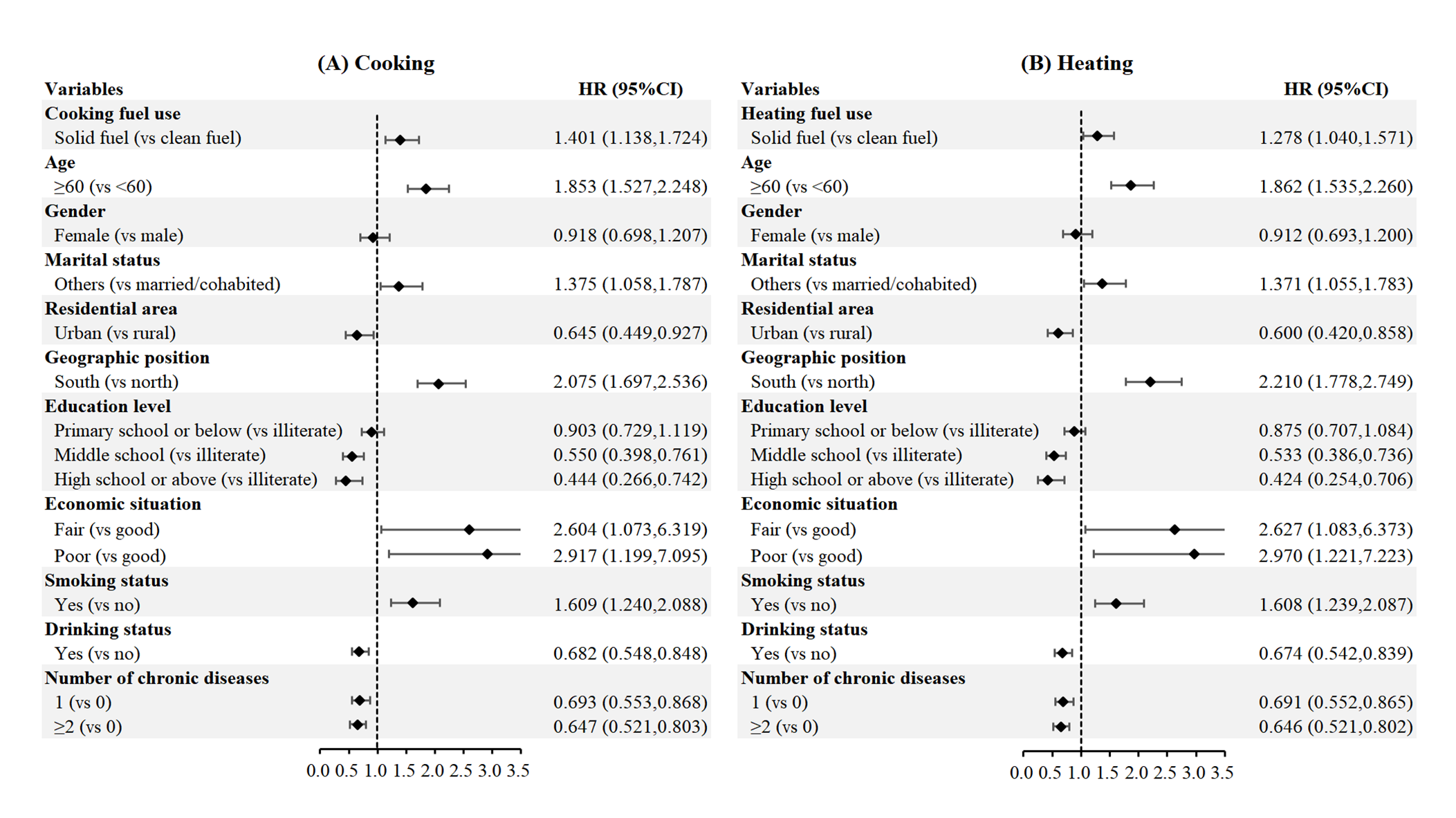
Figure S2** Forest plot of multifactorial Cox analysis between household solid fuel use and sarcopenia.

Abbreviations: HR, hazard ratio; CI, confidence interval.
